# Evaluation of the recurrence and fertility rate following salpingostomy in patients with tubal ectopic pregnancy

**DOI:** 10.1186/s12884-021-04299-y

**Published:** 2022-01-03

**Authors:** Tahereh Poordast, Zahra Naghmehsanj, Razie Vahdani, Shaghayegh Moradi Alamdarloo, Mohammad Ali Ashraf, Almtaj Samsami, Fatemeh Sadat Najib

**Affiliations:** 1 Infertility Research Center, Department of Obstetrics and Gynecology, Shiraz Medical School, Shiraz University of Medical Sciences, Shiraz, Iran; 2 Student Research Committee, Shiraz University of Medical Sciences, Shiraz, Iran; 3 Maternal-Fetal Medicine Research Center, Shiraz University of Medical Sciences, Shiraz, Iran; 4 OB & GYN Department, Shiraz University of Medical Sciences, Shiraz, Iran

**Keywords:** Ectopic pregnancy, Laparoscopy salpingostomy, Fertility, Recurrence

## Abstract

**Background:**

Ectopic pregnancy is one of the leading causes of pregnancy-related mortality; the treatment strategies associated with this condition entail complications, such as recurrence of ectopic pregnancy or infertility. The objective of this study was to evaluate the recurrence and fertility rate after salpingostomy in patients with tubal ectopic pregnancy.

**Methods:**

This cross-sectional retrospective study was conducted at four referral centers of Obstetrics and Gynecology, under the supervision of Shiraz University of Medical Sciences (Iran). The medical records of 125 patients with tubal pregnancy were reviewed. These patients underwent laparoscopic salpingostomy from April 2009 to March 2016.Data on maternal age, BMI, history of previous EP, genital tract infection, IUD insertion, history of previous surgery, and infertility were further obtained. The patients were followed up for approximately 1 to 7 years. The recurrence of EP and subsequent pregnancy rate were assessed during the follow-up period.

**Results:**

There was no statistically significant relationship between post-salpingostomy recurrence and maternal age, previous abdominopelvic surgery, and history of infertility(*P* = .425); however, the post-salpingostomy recurrence of EP was correlated with BMI (*P* = 0.001), previous history of EP (P = 0.001), genital tract infection (P = 0.001), and IUD insertion (*P* = 003). Among 95 women who had no contraception, pregnancy occurred in 51 cases (53.6%) and recurrence of EP was observed in 16 patients (12.8%).

**Conclusions:**

Our results suggest that salpingostomy is a safe method with a low risk of recurrence and good fertility outcomes for women who consider future pregnancy.

## Background

In ectopic pregnancy (EP), a fertilized ovum is implanted outside the uterine cavity [1]. EP occurs in about 2% of pregnancies [2] and is one of the leading causes of pregnancy-related death [3, 4]. The ampullary part of the fallopian tube is the most common site of EP implantation [5]. Other sites, including ovary, cervix, cesarean scar, and abdominal cavity, have been reported in several studies [6].

The exact etiology of EP is unclear, but it may be caused by impaired tubal transport [7]. Other risk factors that affect the function or structure of fallopian tubes include pelvic inflammatory disease, tubal surgery, prior tubal EP, smoking, and in-vitro fertilization [8].

Although it may be asymptomatic, EP frequently presents with pain and vaginal bleeding in the first trimester of pregnancy [9, 10]; however,6-10% of patients present with ruptured EP and unstable hemodynamics [11]. Transvaginal sonography is a standard method for EP diagnosis [12], and the early detection owing to the improvements in this imaging modality has reduced mortality and morbidity [13].

Treatment of EP has different strategies, such as expectant management (following the *β*-human chorionic gonadotropin (β-hCG) until its return to the normal level), medical treatment (methotrexate), and surgery [14, 15]. The management choice depends on the serum level of B-hCG, EP site, history of the previous EP, desire for a future pregnancy, and surgical skills [14].

Radical method (removing the fallopian tube) and conservative technique (incising the fallopian tube to remove EP) by either laparotomy or laparoscopy are the two most common surgical approaches [6]. Laparoscopy has become the preferred surgical method owing to its lower cost and operative risk [6]. Laparoscopic conservative surgery includes salpingostomy, salpingotomy, segmental tubal resection, and anastomosis [16]. Salpingectomy is a definitive surgical treatment for ectopic pregnancy, but salpingostmy is the preferred one because of the patients’ desire for subsequent pregnancies. Conservative methods like laparoscopic salpingostomy entail certain complications, such as persistent trophoblastic tissue or recurrence of EP in the operated tube [16]; therefore, it is necessary to glean more data as to the efficacy of this procedure. The aim of this study was to assay the clinical characteristics associated with the recurrence of ectopic pregnancy following salpingostomy and its influence on ongoing pregnancy.

## Methods

This cross-sectional retrospective study was conducted at four referral centers of Obstetrics and Gynecology under the supervision of Shiraz University of Medical Sciences (Iran) (Hazrat-e-zaynab, Shahid Faghihi, Madar-o-koodak and Dena hospital). The medical records of all patients with tubal pregnancy who underwent laparoscopic salpingostomy from April 2009 to March 2016 were reviewed. Demographic data such as maternal age, BMI, history of the previous EP, genital tract infection, IUD insertion, history of previous surgery, and infertility were extracted from the patient’s available records.

Indications for salpingostomy included hemodynamic stability, unruptured tube, contraindications to methotrexate or failed medical treatment. During surgery, the appearance of contralateral tube was checked.

The exclusion criteria were 1) patients with non-tubal ectopic pregnancy, 2) those treated with salpingectomy or other conservative surgical management (such as laparoscopic milking) or treated by methotrexate, and 3) subjects who were unwilling to participate, refused to answer the question, or had incomplete data in the hospital chart.

After surgery, patients were followed by serial serum B-HCG level until it became undetectable. The patients were followed up for approximately 1 to 7 years, during which period the recurrence of EP and the subsequent pregnancy rate were assessed (Only the first IUP or the first recurrence was used for this analysis).

Women reporting no desire of subsequent pregnancy after the salpingostomy, and who, therefore, did not actively try to conceive or were using a contraceptive method were excluded. Also, patients who had received medical treatment with methotrexate after surgery, patients with male factor infertility, those who had hospitalization due to PID (pelvic inflammatory disease) after surgery, and patients that their age become more than 45 years in the follow-up period were excluded. These strict exclusion criteria were considered to reduce the likelihood of bias.

Patients who actively tried to conceive after the salpingostomy and who accepted to be involved in the study were included.

Finally, 125 patients were assessed in this study, the demographic data such as BMI, reassessed in the follow up period, which had no significant changes.

Data regarding the past medical history and demographics were extracted from hospital paper records to decrease recall bias. Also, inquired future outcomes (the recurrence of EP and the subsequent pregnancy) were rechecked with hospital records to decrease the chance of bias.

The study protocol was approved by the institutional Ethics Committee (code: IR.SUMS.MED.REC.1395.s72). Data were analyzed using SPSS version 19. Pearson correlation was used to determine the correlations and *P* value < 0.05 was considered as statistically significant.

## Results

An overall of 3434 patients who were diagnosed with EP in our centers from 2009 to 2016 were enrolled in the present study. Among them, 2244 (65.34%) patients were treated with non-surgical methods and 1190 patients had surgery. Salpingectomy and salpingostomy were carried out for 1025 (29.85%) and 165 (4.8%) cases, respectively.

Finally, 165 patients were selected, 40 of whom were excluded because they were not willing to answer the questions, gave the wrong phone numbers, or did not answer their phones.

Among the 125 patients, the mean age was 26.496 ± 5.3 years (maximum = 40 and minimum = 17) and the mean BMI was 24.46 ± 3.6 kg/m^2^ (maximum = 37.1 and minimum = 15.87). The mean B-HCG level was 2706 ± 3.39 mIU/ml (112-19,925) and the mean size of the ectopic mass was 8.8 ± 6.8 cm^2^ (1-35) at the time of admission.

During laparoscopy, the appearance of bilateral tubes was checked, and salpingostomy was done as a treatment for unruptured ectopic pregnancies.

66 (528%) cases developed ectopic pregnancy in the right adnexa and 59 (47.2%) in the left adnexa (Table 1). In all cases, contralateral tube appeared normal

**Table 1:** The baseline characteristics of the patients who had undergone salpingostomy

Variable	All cases (*n* = 125)
Age, year, mean ± SD	26.49 ± 5.3
BMI, Kg/m2,mean ± SD	24.49 ± 3.6
Site of EP:	
RT site	66(52.8%)
LT site	59(47.2%)

The complication of salpingostomy in persistent trophoblastic disease was detected in 5-15%of cases in some studies (5). In our study, the patients were followed up based on B-HCG level, none presented with persistent diseases, and there was no need for treatments, such as methotrexate or second surgery.

Also, no surgical complications were reported after salpingostomy in our patients.

The evaluated risk factors for postoperative recurrence were maternal age, BMI, history of the previous EP, genital tract infection, IUD consumption (prior to salpingostomy), history of previous surgery, and infertility. History of infertility was observed in 32% of the patients; furthermore, previous pelvic surgery, genital tract infection, previous EP, and IUD insertion were found in 28, 18.4, 16.8, and 10.4% of the cases, respectively. Those who had a previous history of EP were treated with non-surgical methods (25% with methotrexate and 75% without medication) only with expectant management.

There was no statistically significant correlation between maternal age and post-salpingostomy recurrence (*P* = .425); however, as seen in Table 2, this recurrence was strongly correlated with BMI (*P* = 0.001), previous history of EP (P = 0.001), genital tract infection (P = 0.001), and IUD insertion (*P* = 0.03).

**Table 2:** The risk factors associated with therecurrence of EP after salpingostomy

Variable	Recurrence group(*n* = 16)	Non-recurrence group(*n* = 109)	P value
Age, year, mean ± SD	27.43	26.37	0.425
BMI, Kg/m2,mean ± SD	27.14	24.06	0.001
Positive history of prior EP(n)	8	13	< 0.001
Positive history of infections(PID)	8	15	< 0.001
Positive history of IUD insertion	5	8	0.003

Multiple logistic regression test was done, and the results showed that for every unit of BMI increase, the chance of recurrence rose by 20%.

The chances of recurrence in patients with a positive history of EP were 9.75 times higher than those with no such history (odds ratio = 9.75).

No statistical correlation was found between recurrence of EP post-salpingostomy and previous abdominopelvic surgery (sig = 0.757) or history of infertility (sig = 0.945). Among the 95 women who had no contraception, pregnancy occurred in 51 cases (53.6%) and EP recurrence was seen in 16 cases (12.8%), 0.33% at the site of salpingostomy and0.67% in contralateral tube. All the patients with recurrence (16 people) had at least one risk factor in their life (Table 3).

**Table 3:** Pregnancy rate in salpingostomy cases during the follow-up period

Recurrence of EP^a^ (n)	Pregnancy (n)	ART^b^	(n)	(%)	Contraception	(n)	(%)
NO (109)	NO (59)	NO	41	32.8	NO	37	29.6
		YES	18	14.4	yes	22	17.6
	YES (50)	NO	34	27.2	NO	42	33.6
		YES	16	12.8	YES	8	6.4
YES (16)	NO (15)	NO	7	5.6	NO	15	12
		YES	8	6.4	YES	0	0
	YES (1)	NO	1	0.8	NO	1	0.8
		YES	0	0	YES	0	0

^a^EP = ectopic pregnancy

^b^ART = assisted reproductive technology

## Discussion

Among the several well-known factors that augment the risk of ectopic pregnancy, mention can be made of pelvic inflammatory disease (*Chlamydia trachomatis* or Neisseria gonorrhea) and tubal surgery (salpingostomy, neosalpingostomy, fimbrioplasty, tubal re-anastomosis, and lysis of peri-tubal or peri-ovarian adhesions) [17]. Although tubal ligation effectively prevents pregnancy, if pregnancy does occur, it is probably ectopic. Krik et al. reported that adnexal surgery and previous pelvic inflammatory disease were the most important risk factors associated with EP [18]. In the present study, positive history of genital tract infection was reported in 18.4% of patients, and a history of prior abdominopelvic surgeries, such as cesarean section, appendectomy, tuboplasty, and diagnostic laparoscopy, was found in 28%.

Bangsgaard observed that a history of the previous EP increased the risk of future EP up to 10–25% [17]. Chandrasekhar found that the odds ratio of EP was 12.5 after one previous ectopic pregnancy and 76.6after two previous EP s[19]. These findings are in the same line as our study, where12.8% of all cases developed EP after at least one previous ectopic pregnancy, and the chances of recurrence in subjects with a history of previous EP were9.7 times more than those with no such history (odds ratio: 9.7).

Oron et al. showed that the risk of EP using norgestrel-containing IUD was lower than that with a copper IUD. This might be attributed to the reduced overall pregnancy rate in women using norgestrel-containing IUDs [20]. Based on our results,10.4% of women used copper IUD, and there was a significant correlation between IUD (*P* value = 0.003) and EP recurrence following salpingostomy.

The risk of EP increased with the rise in maternal age over 35 years. This association may be ascribed to most other risk factors related to an increase in age [9]. History of infertility is the other risk factor for EP; Krik et al. revealed that tubal infertility, non-tubal infertility, and in vitro fertilization (IVF) treatment correlated with EP ris k[18]. About 32% of our patients were infertile, and no correlation was found between infertility and EP recurrence. A few studies have discussed BMI as a risk factor for EP, which is consistent with the current study, in which a correlation was observed between BMI and the recurrence of post-salpingectomy EP (*P* value = 0.001); this indicates that more studies should be conducted concerning this topic.

Langer et al. reported that the EP recurrence rate was 7% if the contralateral tube was normal and 18-25% if the opposite tube was abnormal or absent [21]. In a prospective study, Olofsson et al. reported that the pregnancy rates were not significantly different before and after salpingostomy, salpingectomy, or methotrexate treatment [22].

Agdi and Tulandi reviewed the literature on different treatments for tubal pregnancy. Among patients who attempted to conceive, the subsequent intrauterine pregnancy (IUP) rate was 61.4% after salpingostomy, 38.1% following partial or total salpingectomy, and 54% after treatment with methotrexate. The recurrent ectopic pregnancy rates after salpingostomy, salpingectomy, and methotrexate treatment were 15.4, 9.8, and 8%, respectively [23]. Ding et al. found no difference in the rate of intrauterine pregnancy before and after salpingostomy or salpingectomy. However, the rate of recurrent ectopic pregnancy was slightly higher after salpingostomy, particularly if the affected tube was diseased [24].

Our study showed that among the women who attempted to conceive (95 cases), 53.68% had IUP after salpingostomy, which seems to be a good result in comparison with other studies. Finally, the recurrence of EP after salpangostomy in our study was estimated at about 12.8%, which is similar to previous studies.

## Conclusions

Our results suggest that salpingostomy is a safe method with a low recurrence risk and good fertility outcomes for women who have a desire for future pregnancies.

## Data Availability

The datasets used and analyzed during the current study are available from the corresponding author on reasonable request.

## Abbreviations

EP: Ectopic pregnancy
β-hCG: β-human chorionic gonadotropin
IVF: in vitro fertilization
IUP: intrauterine pregnancy
